# Two New Bromophenols with Radical Scavenging Activity from Marine Red Alga *Symphyocladia latiuscula*

**DOI:** 10.3390/md11030842

**Published:** 2013-03-13

**Authors:** Xiuli Xu, Liyuan Yin, Lijie Gao, Junhai Gao, Junhui Chen, Jingxi Li, Fuhang Song

**Affiliations:** 1 School of Ocean Sciences, China University of Geosciences, Beijing 100083, China; E-Mails: yinliyuan999@sina.com (L.Y.); gaolijie2012@gmail.com (L.G.); gaojunhai2012@gmail.com (J.G.); 2 Marine Ecology Research Center, First Institute of Oceanography, State Oceanic Administration, Qingdao 266061, China; E-Mails: jhchen@fio.org.cn (J.C.); jxli@fio.org.cn (J.L.); 3 Chinese Academy of Sciences Key Laboratory of Pathogenic Microbiology and Immunology, Institute of Microbiology, Chinese Academy of Sciences, Beijing 100190, China

**Keywords:** red alga, *Symphyocladia latiuscula*, bromophenol

## Abstract

Chemical investigation of a Chinese collection of marine red alga *Symphyocladia latiuscula* yielded two new highly brominated phenols. The structures of the new compounds were elucidated by detailed spectroscopic analysis, including HRMS, 1D and 2D NMR and MS methods. Compounds **1** and **2** were evaluated for radical scavenging capability by 1,1-diphenyl-2-picrylhydrazuyl (DPPH) radical with the IC_50_ value of 14.5 and 20.5 μg/mL, respectively.

## 1. Introduction

**Figure 1:**
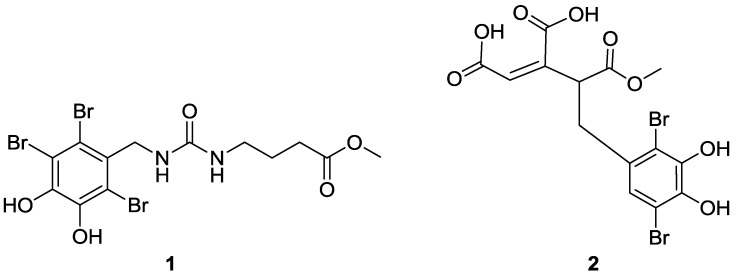
Structures of compounds **1** and **2**.

*Symphyocladia latiuscula* (Harvey) Yamada is a marine red alga distributed along the coasts of Northern China, Korea, and Japan [1]. This red alga is a rich source of bromophenols with high chemical diversity and various biological activities. Previous chemical studies on this species have resulted in the characterization of 25 monoaryl and diaryl bromophenols with a variety of bioactivities, such as antibacterial [2,3], antifungal [4,5], free-radical-scavenging [6,7], aldose reductase inhibitory [8], antiviral [9], anticancer [10] and Taq DNA polymerase inhibitory activities [11]. During the course of our continuing search for new biologically active bromophenols from this marine red alga, by mass spectrum guided fractionation, two new bromophenols (**1** and **2**) with radical scavenging activity were characterized (Figure 1). Herein, we report the isolation, structure elucidation and bioactivity evaluation of these bromophenols.

## 2. Results and Discussion

**Figure 2:**
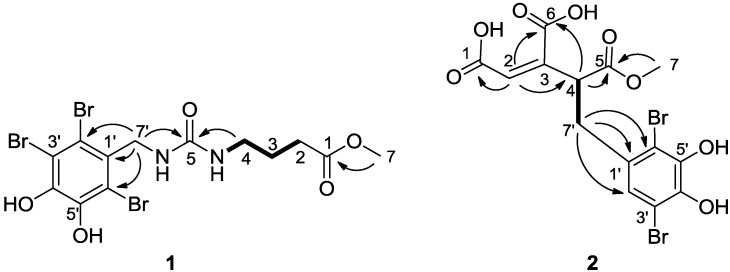
Key HMBC correlations (H→C) and ^1^H–^1^H COSY (bold line) for compounds **1** and **2**.

Compound **1** was obtained as a light brown amorphous powder. The positive ESIMS of **1** gave a pseudo molecular ion peak cluster for a tribrominated molecule at 517/519/521/523(1:3:3:1) [M + H]^+^. The molecular formula was determined to be C_13_H_15_Br_3_N_2_O_5_ by analysis of its HRESIMS (*m/z* 516.8613 [M + H]^+^). The ^1^H NMR spectrum of **1** showed three multiplets assignable to a 1,3-disubstituted propane unit at δ 2.99 (2H, quartet, *J* = 6.6 Hz, H_2_-4), 1.60 (2H, quintet, *J* = 6.6 Hz, H_2_-3), and 2.28 (2H, t, *J* = 6.6 Hz, H_2_-2), one doublet sp^3^ methylene at δ 4.51 (2H, d, H-7′) and an ester methoxyl singlet at δ 3.58 (3H, s, *O*CH3), and two exchangeable broad triplets assigned to amino protons at δ 5.85 (1H, brt, *J* = 4.8 Hz, N′-H) and 5.84 (1H, brt, *J* = 6.6 Hz, N–H). The ^13^C NMR data for **1** revealed the carbon signals associated with the above structural units (Table 1) as well as a set of resonances for the 2,3,6-tribromo-4,5-dihydroxybenzyl unit [2,3,4,5,6,7,8] and two additional signals associated to ester carbonyl carbon at δ 173.2 (C-1) and one sp^2^-hybridized quaternary carbon at δ 157.4 (C-5). The NMR signals of protons and corresponding carbons were assigned by the ^1^H–^1^H COSY and HSQC experiments (Table 1). The structure of **1** was unambiguously established by ^1^H−^1^H COSY, HSQC, and HMBC experiments. In the HMBC spectrum, the correlations (Figure 2) from H_2_-7′ to C-1′, C-2′, and C-6′, in combination with chemical shift values of brominated quaternary carbons (δ < 120) and oxygenated quaternary carbons (δ > 140), demonstrated the existence of the 2,3,6-tibromo-4,5-dihydroxybenzyl unit in **1**. The HMBC correlations from H_2_-2, H_2_-3, and the methoxyl protons to C-1, H_2_-2 and H_2_-3 to C-4, H_2_-4 to C-5, together with the ^1^H–^1^H COSY signals between H_2_-4 and H–N and between H_2_-3 and H_2_-2 and H_2_-4 and between H_2_-7′ and H-N′, revealed the presence of a methyl γ-ureidobutyrate moiety in **1**. In addition, HMBC correlations from H_2_-7′, H-N′, H-N and H_2_-4 to C-5 revealed that C-5 connected across N to C-7 and across N′ to C-7′. Therefore, **1** was determined as methyl *N*′-(2,3,6-tibromo-4,5-dihydroxybenzyl)-γ-ureidobutyrate. It is very interesting that the similar compound methyl *N*′-(2,3-dibromo-4,5-dihydroxybenzyl)-γ-ureidobutyrate was isolated from the same family marine red alga *Rhodomela confervoidesc* [12]. The specific halogenase may play an important role in the biosynthesis of these bromophenols.

**Table 1:** NMR data for compounds **1** (600 MHz, DMSO-*d*_6_) and **2** (600 MHz, Methanol-*d*_4_).

Compound 1				Compound 2		
pos	C mult	(*J* in Hz)	HMBC ^a^	C mult	(*J* in Hz)	HMBC ^a^
1	173.2, C			168.4, C		
2	30.7, CH_2_	2.28, t (6.6)	1, 3, 4	131.5, CH	6.76, s	
3	25.5, CH_2_	1.60, quint (6.6)	1, 2, 4	144.2, C		
4	38.5, CH_2_	2.99, quart (6.6)	2, 3, 5	44.2, CH_2_	5.06, dd (10.8, 4.2)	2, 3, 5, 6, 7′
N–H		5.84, brt (6.6)				
5	157.4, C			174.2, C		
6				169.1, C		
*O*Me	51.2, CH_3_	3.58, s	1	52.7, CH_3_	3.68, s	5
1′	129.7, C			132.0, C		
2′	116.8, C			126.3, CH	6.73, s	3′, 4′, 6′, 7′
3′	113.8, C			109.6, C		
4′	144.7, C			143.7, C		
5′	143.7, C			145.4, C		
6′	113.7, C			113.5, C		
7′	46.7, CH_2_	4.51, d (4.8)	1′, 2′, 6′	36.9, CH_2_	3.60, dd (13.8, 4.2 )	3, 4, 5, 1′, 2′, 6′
			5		2.98, dd (13.8, 10.8 )	3, 4, 5, 1′, 2′, 6′
N–H′		5.85, brt (4.8)				

^a^ HMBC correlations, optimized for 8 Hz, are from proton(s) stated to the indicated carbon.

Compound **2** was obtained as a light brown amorphous powder. The positive ESIMS of **1** gave a pseudo molecular ion peak cluster for a tribrominated molecule at 467/469/471(1:2:1) [M + H]^+^. The molecular formula was determined to be C_14_H_12_Br_2_O_8_ by analysis of its HRESIMS (*m/z* 466.8985 [M + H]^+^). The ^1^H NMR spectrum of **2** displayed two alkenyl protons at δ 6.76 (H, s, H-2) and 6.73 (H, s, H-2′), one sp^3^methylene at δ 3.60 (1H, dd, 13.8, 4.2, H-7′a) and 2.98 (1H, dd, 13.8, 10.8, H-7′b), one doublet doublets at δ 5.06 (1H, dd, 10.8, 4.2, H-4) and an ester methoxyl singlet at δ 3.68 (3H, s, *O*CH3). The ^13^C NMR data for **2** revealed a *cis*-aconitic acid methyl ester moiety which was reported from the same species in our previous studies [4], two oxygenated quaternary at downfield (δ > 140) and two brominated quaternary carbons at high field (δ < 120). The *cis*-aconitic acid methyl ester moiety was confirmed by the HMBC cross peaks from H-2 to C-1, C-4 and C-6, from H-4 to C-5 and C-6 and from H_3_-7 to C-5. Meanwhile, the HMBC correlations from H-2′ (δ 6.73, s) to C-3′, C-4′, C-6′ and -7′ and from H_2_-7′ to C-1′, C-2′ and C-6′ revealed that this compound contains a 3,6-dibromo-4,5-dihydroxybenzyl unit. Finally, the connection between C-4 and C-7′ was assigned by the COSY signals between H-4 and H-7′a and 7′b. The ROESY correlation between H-2 and H-4 established the *Z* configuration about ∆^2,3^. The absolute configuration of C-4 was not assigned. Although methanol was used during the isolation, only mono-methyl ester of the tri-acid compound was detected from these samples. These data indicated that compound **2** could be a natural product. Microbial reductive dechlorination of polychlorinated biphenyls can occur in aquatic sediments [13], which suggested that the 2′-debrominated analogues **2** might be derived from the reductive debromination of the hexa-substituted compound. 

Compounds **1** and **2** were evaluated for radical scavenging capability on 1,1-diphenyl-2-picrylhydrazuyl (DPPH) radical. Compounds **1** and **2** exhibited moderate radical scavenging ability with IC_50_ value of 14.5 and 20.5 μg/mL, respectively. The IC_50_ of positive control vitamin C is 7.82 μg/mL.

## 3. Experimental Section

### 3.1. General Experimental Procedures

NMR spectra were recorded on a Varian Inova 600 MHz spectrometer at 600 MHz for ^1^H and 125 MHz for ^13^C in DMSO-*d*_6_ and Methanol-*d*_4_ using solvent signals (DMSO: δ_H_ 2.50/δ_C_ 39.51; Methanol: δ_H_ 3.31/δ_C_ 49.15) as reference; the coupling constants were in Hz. ESIMS spectra were recorded with a ABI Mariner ESI-TOF. Column chromatography was performed with silica gel (200–300 mesh, Qingdao Haiyang Chemical Factory) and Sephadex LH-20 (Pharmacia Co., Sweden) columns. HPLC was performed using an Agilent 1100 Series separations module equipped with Agilent 1100 Series diode array detector and performed using an Agilent Zorbax Eclipse XDB-C8 (5 μm) semipreparative column (9.4 × 250 mm). 

### 3.2. Algal Material

*Symphyocladia latiuscula* was collected on the coast of Qingdao, Shandong Province, China, in May 2004. The specimen identification was verified by Dr. Kui-Shuang Shao (Institute of Oceanology, Chinese Academy of Sciences, Qingdao 266071, China). A voucher specimen (No. 2004X16) was deposited at the Herbarium of the Institute of Oceanology, Chinese Academy of Sciences, Qingdao 266071, China.

### 3.3. Extraction and Isolation

The air-dried red alga Symphyocladia latiuscula (4.3 kg) was extracted with 95% EtOH at room temperature (3 × 72 h). After the solvent was removed under reduced pressure at <40 °C, a dark residue (610 g) was obtained. The residue was partitioned between EtOAc and H_2_O and the EtOAc-soluble partition (320 g) was chromatographed over silica gel, eluting with a gradient of 0%–100% Me_2_CO/petroleum ether [4]. The fraction eluted by 30% Me_2_CO/petroleum ether was further fractionated over Sephadex LH-20 using petroleum ether–CHCl_3_–MeOH (5:5:1) to afford 18 fractions. The ninth fraction from the LH-20 column was further fractionated by ODS column, which was eluted with a stepwise gradient of 0%–100% MeOH/H_2_O to afford 11 subfractions. The forth ODS subfraction was subjected to HPLC fractionation (Zorbax Eclipse XDB-C8 5 μm 250 × 9.4 mm column) to yield compound **1**. The 13th fraction from the LH-20 column was further fractionated by ODS column, which was eluted with a stepwise gradient of 0%–100% MeOH/H_2_O to afford 11 subfractions. The third ODS subfraction was subjected to HPLC fractionation (Zorbax Eclipse XDB-C8 5 μm 250 × 9.4 mm column) to yield compound **2**.

Compound **1**: Light brown amorphous powder; IR ν_max_ 3335, 2953, 1702, 1627, 1548, 1452, 1398, 1365, 1168, 1117, 1067, 1031, 968, 942, 910, 882, 831, 766, 702 cm^−1^；^1^H and ^13^C NMR data, see Table 1; ESIMS *m/z* 517/519/521/523(1:3:3:1) [M + H]^+^; HRESIMS *m/z* by analysis of its HRESIMS (*m/z* 516.8613 [M + H]^+^) (calcd for C_13_H_16_^79^Br_3_N_2_O_5_, 516.8604). 

Compound **2**: Light brown amorphous powder; [α]^20^_D_ +10.0 (*c* 0.05, MeOH); IR ν_max_ 3367, 3296, 2954, 2615, 1766, 1715, 1643, 1568, 1472, 1439, 1423, 1264, 1137, 1018, 933, 910, 816, 643, cm^−1^; ^1^H and ^13^C NMR data, see Table 1; ESIMS *m/z* 467/469/471(1:2:1) [M + H]^+^; HRESIMS *m/z* 466.8985 [M]^+^ (calcd for C_14_H_13_^79^Br_2_O_8_, 466.8972).

### 3.4. Scavenging Ability on 1,1-Diphenyl-2-picrylhydrazuyl (DPPH) Radical

Each power (0.1–20 mg/mL, 4.0 mL) in deionized water was mixed with 1.0 mL of methanolic solution containing DPPH (Sigma) radicals, resulting in a final concentration of 0.2 mM DPPH. The mixture was shaken vigorously and left to stand for 30 min in the dark, and the absorbance was then measured at 517 nm against a blank [14]. The scavenging ability was calculated as follows:

scavenging ability (%) = [(A517 of control − A517 of sample)/A517 of control] × 100 (1)

Vitamin C was used for positive control.

## 4. Conclusions

*Symphyocladia latiuscula* is a rich source of bromophenols with specific subunit of 2,3,6-tribromo-4,5-dihydroxybenzene. During the course of our systematic search for new biologically active bromophenols from this marine red alga, two new bromophenols (**1** and **2**) with radical scavenging activity were characterized by mass spectrum guided fractionation. Compounds **1** and **2** exhibited radical scavenging capability on 1,1-diphenyl-2-picrylhydrazuyl (DPPH) radical with the IC_50_ value of 14.5 and 20.5 μg/mL, respectively. 
